# Applying Human ADAR1p110 and ADAR1p150 for Site-Directed RNA Editing—G/C Substitution Stabilizes GuideRNAs against Editing

**DOI:** 10.3390/genes8010034

**Published:** 2017-01-14

**Authors:** Madeleine Heep, Pia Mach, Philipp Reautschnig, Jacqueline Wettengel, Thorsten Stafforst

**Affiliations:** Interfaculty Institute of Biochemistry, University of Tübingen, Auf der Morgenstelle 15, 72076 Tübingen, Germany; mad.heep@web.de (M.H.); pia.mach@student.uni-tuebingen.de (P.M.); philipp.reautschnig@uni-tuebingen.de (P.R.); jacqueline.wettengel@uni-tuebingen.de (J.W.)

**Keywords:** site-directed RNA editing, ADAR, guideRNA, genetic disease, RNA repair

## Abstract

Site-directed RNA editing is an approach to reprogram genetic information at the RNA level. We recently introduced a novel guideRNA that allows for the recruitment of human ADAR2 to manipulate genetic information. Here, we show that the current guideRNA design is already able to recruit another human deaminase, ADAR1, in both isoforms, p110 and p150. However, further optimization seems necessary as the current design is less efficient for ADAR1 isoforms. Furthermore, we describe hotspots at which the guideRNA itself is edited and show a way to circumvent this auto-editing without losing editing efficiency at the target. Both findings are important for the advancement of site-directed RNA editing as a tool in basic biology or as a platform for therapeutic editing.

## 1. Introduction

**Figure 1:**
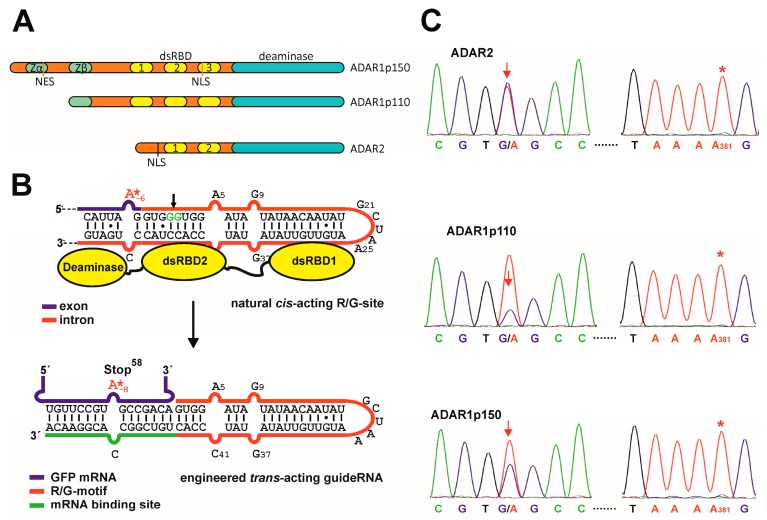
Site-directed RNA editing with R/G-guideRNAs. (**A**) Scheme of the three human ADARs used in this study; (**B**) Design principle: the R/G-guideRNAs have been developed as trans-acting guideRNA from the natural cistronic R/G-motif of the GluR2 transcript. The binding sites of the dsRBDs (dsRNA-binding domains) of ADAR2 are indicated; (**C**) Sanger sequencing of the editing experiments when applying the R/G-guideRNA with ADAR1p110 or p150 compared to ADAR2 in the repair the W58x codon in eGFP. Shown is the sequence around the editing site (arrow) and around a typical off-target site, A381 (*). For full Sanger sequences see Figures S2 and S3.

Site-directed RNA editing is a method to recode genetic information at the RNA level [1]. The approach is based on the enzymatic conversion of adenosine (A) to inosine (I). Inosine is interpreted as guanosine in many biochemical processes including translation, thus A-to-I RNA editing allows the recoding of amino acids, splice elements, miRNAs, and miRNA binding sites among others [2]. We and others have recently developed methods, called site-directed RNA editing, that employ engineered deaminases in combination with short guideRNAs to recode single adenosine bases at specific sites in any user-defined transcript [3,4]. Due to the usage of guideRNAs, the target selection and specificity is easily and rationally programmed based on simple Watson–Crick base pairing rules. We and others have shown the functioning of site-directed editing in the PCR tube, in human cell lines and even in simple organisms for the repair of reporter genes, but also disease-relevant genes like CFTR [4,5,6]. However, with respect to the ease of the procedure and in particular with respect to application in medicine, the requirement for the expression of an engineered deaminase is a limiting factor. Hence, we have recently developed a guideRNA architecture derived from the natural R/G-site of the GluR2 transcript that enables the recruitment of human ADAR2 for site-directed RNA editing (Figure 1B) [7]. Applying such guideRNAs allows for the editing of endogenous housekeeping genes by expression of a guideRNA only. Furthermore, we demonstrated the recoding of a nonsense mutation in PINK1 to a level sufficient to rescue the mitophagy phenotype that links dysfunctional PINK1 to the etiology of Parkinson’s disease.

Human cells express three different forms of ADAR (adenosine deaminase acting on RNA), called ADAR1, 2, and 3, which are potentially re-addressable for site-directed RNA editing (Figure 1A) [2,8]. Besides ADAR2, the recruitment of ADAR1 is particularly interesting. On one hand ADAR1 is highly active for the deamination reaction and has shown to exhibit a slightly different substrate preference [9]. On the other hand, ADAR1 is more ubiquitously and to a higher level expressed as compared to ADAR2 [10], which is mainly expressed in neurons and which is believed to be exclusively localized to the nucleus [11]. ADAR1 is expressed in two isoforms, an interferon-inducible long form, called p150, and a constitutive, short form, called p110 (Figure 1A). The long form (p150) comprises an additional N-terminal stretch containing the Z-DNA/Z-RNA binding domain Zα and a nuclear export signal (NES) [12]. Due to the latter, ADAR1p150 is mainly found in the cytosol. ADAR1 and ADAR2 have distinct but also overlapping substrates. Both are essential as we know from knockout studies in mice [13,14,15]. Alteration of RNA editing is linked to various neurological diseases including behavioral disorders, epilepsy, and the Prader-Willi syndrome [16,17,18,19]. Mutations in ADAR1 are linked to the Aicardi-Goutieres syndrome [20], an autoimmune disorder, and others [21]. Both, hyper- [22] and hypoediting [23] have been associated with cancer [24,25,26].

Here, we demonstrate that a trans-acting guideRNA that has recently been developed for the recruitment of ADAR2 is also capable of recruiting both ADAR1 isoforms. Furthermore, we studied the auto-editing of the guideRNA itself, and present novel guideRNA sequences that are less prone to auto-editing but still allow for the recruitment of ADAR2.

## 2. Materials and Methods

### 2.1. ADAR1-Expressing Cells

The 293 Flp-In T-REx system (Life Technologies) was used for stable integration of a single copy of the cDNA of ADAR1p110 or ADAR1p150 at a genomic FRT-site in engineered 293 cells. Briefly, 4 × 10 ^6^ cells were seeded on a 10 cm dish. After one day, 1 µg of the respective ADAR1-form in a pcDNA5 vector under control of the tet-on CMV promoter, and 9 µg of pOG44 expressing the Flp recombinase were transfected with lipofectamine 2000 (30 µL). One day later, the medium was changed for at least two weeks to selection medium (DMEM, 10% FBS, 100 µg/mL hygromycin B, 15 µg/mL blasticidin S). Cells were kept in selection medium prior to the editing experiment, which was then performed in the absence of antibiotics.

### 2.2. Editing of W58amber eGFP

Protocol for experiments shown in Figure 1 and Figure 3B: The R/G-guideRNAs were subcloned into the pSilencer2.1-U6hygro vector under control of U6 promoter and terminator, the W58x eGFP gene was delivered on a pcDNA3.1 vector as described before [7]. ADAR-expressing 293T cells were cultivated with DMEM, 10% FBS, 1% H/B, 37 °C, 5% CO_2_. Cells (3 × 10^5^/well) were seeded into poly-d-lysin-coated 24-well plates and induced with doxycycline (10 ng/mL). Transfection was carried out 24 h later with lipofectamine 2000, applying 300 ng of W58x GFP and 1300 ng of guideRNA per well. Editing was evaluated 72 h after transfection by isolation and sequencing analysis of the target mRNA. For the latter, total RNA from the cells (NucleoSpin RNA Plus kit, Macherey Nagel, Düren, Germany) was DNaseI-digested, followed by reverse transcription, Taq-PCR amplification, and Sanger sequencing.

### 2.3. Defining Hotspots of Auto-Editing

**Figure 2:**
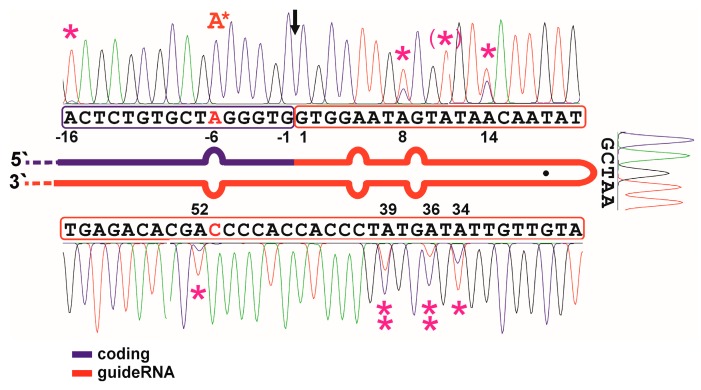
Identification of auto-editing hotspots. An in-vitro transcribed RNA substrate containing a part of the eCFP ORF around the W66x site (blue) in *cis* with an R/G-guideRNA (red) was edited with purified ADAR2 enzyme in a PCR tube. Hotspots for auto-editing have been marked by magenta asterisks; double asterisks mark strongly edited sites; asterisk in brackets mark an editing site only found in a similar experiment inside the cell (see Figure S4). The red A* marks the targeted editing site, full conversion was achieved. The black arrow shows the site where the cistronic motif is cut when the guideRNA is applied in *trans*.

Protocol for the experiment shown in Figure 2: The respective eCFP W66x mRNA containing the guideRNA in *cis* was in-vitro-transcribed with T7 RNA pol as described earlier [7]. ADAR2 was produced and purified as described earlier [7]. Editing was carried out as described earlier [7] with (mRNA) = 25 nM, (ADAR2) = 350 nM, (Mg^2+^) = 3 mM, no heparin, no spermidine, and incubation in editing buffer (12.5 mM Tris, 12.5 mM Tris-HCl, 75 mM KCl, 2 mM DTT) for 180 min while cycling between 30 °C and 37 °C.

### 2.4. Testing Auto-Editing Inside the R/G-Motif for Variants 1–4

**Figure 3:**
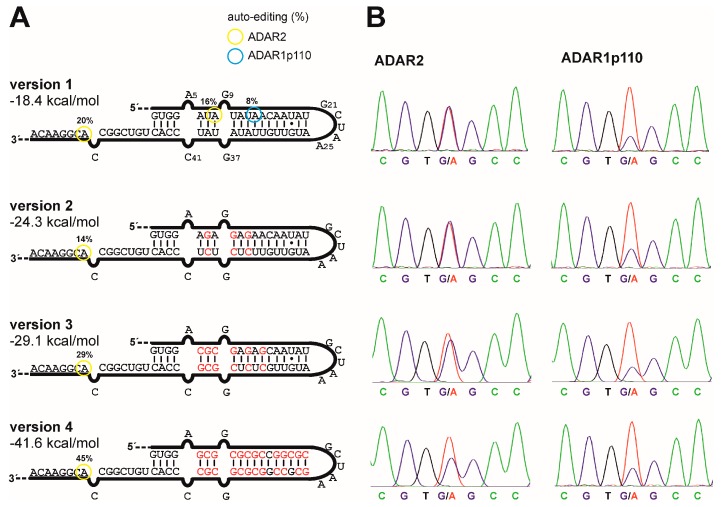
GuideRNA variants that avoid auto-editing. (**A**) Three guideRNA versions with differing degree of G/C substitution were tested for auto-editing. The shown sequence was introduced into the 3′-UTR of the eGFP transcript for easier Sanger sequencing. The folding energies have been estimated using mfold. For ADAR1p150, no auto-editing site was detectable; (**B**) Performance of the three new guideRNA versions for the editing of W58x with ADAR2 or ADAR1p110, respectively, when applied in *trans* in the respective ADAR-expressing cell line. For fluorescence imaging, see Figure S5.

Protocol for experiments shown in Figure 3A: The R/G-guideRNAs were subcloned into the 3′-UTR of wild-type eGFP in a pcDNA3.1 vector. GFP expression served as a transfection control. Editing was carried out in 293T cells under transient expression of one of the respective three ADAR forms from plasmid vectors (CMV promotor). For this, 293T cells (2 × 10^5^/well) were seeded into 24-well plates. Transfection was carried out 24 h later with lipofectamine 2000, applying 300 ng of wt eGFP-guideRNA and 600 ng of the respective ADAR per well. RNA was isolated for sequencing 48 h after co-transfection as described above.

## 3. Results

### 3.1. Site-Directed RNA Editing with ADAR1

In this study, we started from a recently developed guideRNA architecture [7] that is based on the cistronic R/G-motif of the GluR2 transcript (Figure 1B). Compared to the natural R/G-site, the targeted adenosine in the mRNA is put to position −8, thus 2 nt further upstream of the R/G-motif (Figure 1B). We had shown the functioning of the guideRNA by co-transfection of the plasmid-borne guideRNA together with ADAR2 on a plasmid or by transfection of the guideRNA-plasmid into engineered ADAR2-expressing 293 Flp-In cells [7]. To test if the original guideRNA design is able to recruit ADAR1 isoforms, we have now created 293 Flp-In cells that express ADAR1p110 or ADAR1p150 from a single genomic copy under control of a CMV-tet on promoter. In such cell lines, the maximal induction-level of all three ADAR forms was similar and comparable to the level of β-actin mRNA (Figure S1). After induction (24 h) of the respective ADAR with doxycycline, the respective guideRNA construct was co-transfected together with a GFP reporter construct containing a single W58amber missense codon for site-directed repair. 72 h after co-transfection, the editing yield was determined by Sanger sequencing of the RNA (Figure 1), as described earlier [7]. In ADAR2-expressing cells, editing yields around 50% are typically obtained, with no detectable off-target editing in the ORF of the reporter gene [7]. This was again confirmed here (Figure 1C). The editing reaction in ADAR1-expressing cells (Figure 1C) gave slightly reduced yields for the p150 form (35%–40%), and markedly reduced yields (20%) for the p110 form. Again, no off-target editing was detectable for either ADAR1 form in the ORF of the reporter gene. One site, adenosine 381, which was prone to off-target editing under transient overexpression of ADAR2 before [7], is shown in Figure 1C to exemplify the lack of off-target editing in the ORF of eGFP under genomic expression of all three ADARs (see Figures S2 and S3 for full Sanger sequencing traces).

### 3.2. Auto-Editing inside the GuideRNA

The original guideRNA design is relatively AU-rich. Editing could suffer from the auto-editing of the guideRNA itself thus destabilizing the hairpin. We tested this in an initial editing experiment on an in-vitro transcribed RNA substrate that contained the eCFP W66amber sequence in frame with a cis-acting guideRNA inside the PCR tube (Figure 2). Under these conditions, we found five sites in the R/G-motif prone to auto-editing: A8, A14, A34, A36, and A39. All five editing events can be assumed to destabilize the helix. Notably, the editing yields at the sites in the 3′-half of the R/G hairpin were higher compared to those in the 5′-half of the hairpin. We further tested the auto-editing by co-transfection of the same cis-acting CFP-guideRNA construct with ADAR2 in 293T cells (Figure S4). All auto-editing sites were confirmed, the editing yields stayed mostly unchanged, only at A-16 and A34 was the editing yield increased, and, at one new site, A11, auto-editing occurred additionally.

### 3.3. G/C-Substitution inside the R/G-Motif Reduces Auto-Editing

To reduce auto-editing of the guideRNA, 3, 6, or 13 A/U base pairs in the original R/G-motif have been substituted by G/C base pairs, creating variants 2, 3, and 4 (Figure 3A). To assess auto-editing inside these motif variants, we constructed for every guideRNA an eGFP reporter transcript that contains the complete guideRNA in the 3′-UTR of the transcript, thus ca. 550 nt downstream the targeted editing site. This allows for testing the guideRNAs in a situation that resembles the trans-acting guideRNA under concurrent possibility to access auto-editing by Sanger sequencing. Editing was tested by co-transfection of these constructs (300 ng) with each of the three forms of ADAR (600 ng) into 293T cells. Editing in the R/G-motif was analyzed 48 h after co-transfection by RNA isolation and Sanger sequencing. The editing yields inside the R/G-motif are given in color-coded circles in Figure 3A.

Under these assay conditions, the starting guideRNA architecture, version 1, gives only subtle auto-editing, and only at positions A8 by ADAR2 and A13 by ADAR1p110. With ADAR2, we found an additional off-target editing at A52, which is part of the mRNA binding sequence. For ADAR1p150, no auto-editing was detectable. It is reasonable to infer that the 36 bp RNA substrate , given in Figure 2, is much more heavily edited in the R/G-motif compared to the lone-standing, 20 bp R/G-motif, given in Figure 3, as the ADAR enzymes are known to accept dsRNA with ≥30 bp much better than those ≤30 bp [2,11]. When introducing G/C-substitutions into the R/G-motif, no auto-editing was detectable with any of the three enzymes. This was already achieved with only 3 G/C substitutions, which have been chosen in a way to target the main auto-editing sites (A8, A14, A34, A36, and A39) simultaneously with a minimal number of substitutions.

The G/C substitutions do not only remove editable adenosines and put adenosines into contexts non-preferred for editing (e.g., 5′-GA), but they rather increase the free energy gain of folding from −18 kcal/mol up to −42 kcal/mol as estimated by the software mfold [27] (Figure 3A). We tested how the new guideRNA variants 2 to 4 behave compared to the original version 1 for the editing of the reporter W58x eGFP in ADAR2-expressing cells. For versions 2 and 3, virtually no change in the editing yield was observed. Only the highly substituted version 4 showed a reduction in editing yield (Figure 3B, Figure S5). For ADAR1p110, we found a virtually unchanged editing yield with all versions but at comparably low editing efficiency (Figure 3B).

## 4. Discussion

This is the first report about site-directed RNA editing with human ADAR1 isoforms, the constitutive (p110) and the inducible one (p150). As the R/G-guideRNA was developed from a classical ADAR2 substrate for the recruitment of ADAR2, it could be expected that the recruitment of ADAR1 isoforms is less efficient. However, we achieved site-directed RNA editing with the R/G-guideRNAs with both ADAR1 isoforms, in particular with the p150 form; thus, the general strategy is feasible with ADAR1, and our R/G-guideRNA provides a starting point for the development of more appropriate guideRNAs for ADAR1.

Furthermore, we show the first results on auto-editing of the guideRNA itself. We have defined the hotspots for auto-editing and show a simple strategy to circumvent it by minimal substitution in the hairpin secondary structure. The respective optimized guideRNAs are much less auto-edited; however, our results show that a high substitution degree may interfere with editing efficiency at the target. Even though the confinement of auto-editing was not improving the overall editing yield under the given continuous expression of the guideRNA, it could improve editing when drug-like, chemically stabilized R/G-guideRNAs are explored in the future. Notably, when defining the hotspots of auto-editing by applying an artificial eCFP mRNA containing the guideRNA in *cis*, we found full conversion at the targeted adenosine even in cell culture (Figure S4). In contrast, when applying the same guideRNA in *trans*, editing levels above 60% are difficult to obtain. It is likely that there is still space for further optimization of the guideRNA′s design to obtain better editing results with all three enzymes. In summary, both findings are important for the advancement of site-directed RNA editing as a tool in basic biology or as a platform for therapeutic editing [7,28].

## Supplementary Materials

The following are available online at www.mdpi.com/2073-4425/8/1/34/s1, Figure S1: qPCR analysis of ADAR expression in engineered cells, Figure S2: Sanger sequencing trace of editing in ADAR1p110-expressing cells, Figure S3: Sanger sequencing trace of editing in ADAR1p150-expressing cells, Figure S4: Defining auto-editing hotspots in cellular editing; Figure S5: Fluorescence imaging data belonging to experiment shown in Figure 3B.

## References

[1] Vogel P., Stafforst T. (2014). Site-directed RNA editing with antagomir deaminases—A tool to study protein and RNA function. ChemMedChem.

[2] Nishikura K. (2010). Functions and regulation of RNA editing by ADAR deaminases. Annu. Rev. Biochem..

[3] Stafforst T., Schneider M.F. (2012). An RNA–Deaminase conjugate selectively repairs point mutations. Angew. Chem. Int. Ed..

[4] Montiel-Gonzalez M.F., Guillermo I., Yudowski A., Rosenthal J.J.C. (2013). Correction of mutations within the cystic fibrosis transmembrane conductance regulator by site-directed RNA editing. Proc. Natl. Acad. Sci. USA.

[5] Vogel P., Schneider M.F., Wettengel J., Stafforst T. (2014). Improving site-directed RNA editing in vitro and in cell culture by chemical modification of the guideRNA. Angew. Chem. Int. Ed..

[6] Hanswillemenke A., Kuzdere T., Vogel P., Jékely G., Stafforst T. (2015). Site-directed RNA editing in vivo can be triggered by the light-driven assembly of an artificial riboprotein. J. Am. Chem. Soc..

[7] Wettengel J., Reautschnig J., Geisler S., Kahle P. J., Stafforst T. (2016). Harnessing human ADAR2 for RNA repair—Recoding a PINK1 mutation rescues mitophagy. Nucl. Acids Res..

[8] Bass B.L. (2002). RNA editing by adenosine deaminases that act on RNA. Annu. Rev. Biochem..

[9] Schneider M.F., Wettengel J., Hoffmann P.C., Stafforst T. (2004). Optimal guideRNAs for re-directing deaminase activity of hADAR1 and hADAR2 in trans. Nucl. Acids Res..

[10] Picardi E., Manzari C., Mastropasqua F., Aiello I., D’Erchia A.M., Pesole G. (2015). Profiling RNA editing in human tissues: towards the inosinome Atlas. Sci. Rep..

[11] Nishikura K. (2016). A-to-I editing of coding and non-coding RNAs by ADARs. Nat. Rev. Mol. Cell Biol..

[12] Barraud P., Allain F.H.-T. (2012). ADAR Proteins: Double-stranded RNA and Z-DNA binding domains. Curr. Topics Microbiol. Immun..

[13] Higuchi M., Maas S., Single F.N., Hartner J., Rozov A., Burnashev N., Feldmeyer D., Sprengel R., Seeburg P.H. (2000). Point mutation in an AMPA receptor gene rescues lethality in mice deficient in the RNA-editing enzyme ADAR2. Nature.

[14] Hartner J.C., Schmittwolf C., Kispert A., Müller A.M., Higuchi M., Seeburg P.H. (2004). Liver disintegration in the mouse embryo caused by deficiency in the RNA-editing enzyme ADAR1. J. Biol. Chem..

[15] Wang Q., Miyakoda M., Yang W., Khillan J., Stachura D.L., Weiss M.J., Nishikura K. (2004). Stress-induced apoptosis associated with null mutation of ADAR1 RNA editing deaminase gene. J. Biol. Chem..

[16] Morabito M.V., Abbas A.I., Hood J.L., Kesterson R.A., Jacobs M.M., Kump D.S., Hachey D.L., Roth B.L., Emeson R.B. (2010). Mice with altered serotonin 2C receptor RNA editing display characteristics of Prader-Willi syndrome. Neurobiol. Dis..

[17] Maas S., Kawahara Y., Tamburro K.M., Nishikura K. (2006). A-to-I RNA editing and human disease. RNA Biol..

[18] Slotkin W., Nishikura K. (2013). Adenosine-to-inosine RNA editing and human disease. Genome Med..

[19] Silberberg G., Lundin D., Navon R., Öhman M. (2012). Deregulation of the A-to-I RNA editing mechanism in psychiatric disorders. Hum. Mol. Genet..

[20] Rice G.I., Kasher P.R., Forte G.M., Mannion N.M., Greenwood S.M., Szynkiewicz M., Dickerson J.E., Bhaskar S.S., Zampini M., Briggs T.A. (2012). Mutations in ADAR1 cause Aicardi-Goutières syndrome associated with a type I interferon signature. Nat. Genet..

[21] Zhang X.J., He P.P., Li M., He C.D., Yan K.L., Cui Y., Yang S., Zhang K.Y., Gao M., Chen J.J. (2004). Seven novel mutations of the ADAR gene in Chinese families and sporadic patients with dyschromatosis symmetrica hereditaria (DSH). Hum. Mutat..

[22] Chen L., Li Y., Lin C.H., Chan T.H.M., Chow R.K.K., Song Y., Liu M., Yuan Y.F., Fu L., Kong K.L. (2013). Recoding RNA editing of antizyme inhibitor 1 predisposes to hepatocellular carcinoma. Nat. Med..

[23] Shimokawa T., Rahman M.F., Tostar U., Sonkoly E., Ståhle M., Pivarcsi A., Palaniswamy R., Zaphiropoulos P.G. (2013). RNA editing of the GLI1 transcription factor modulates the output of Hedgehog signaling. RNA Biol..

[24] Gallo A. (2013). RNA editing enters the limelight in cancer. Nat. Med..

[25] Paz-Yaacov N., Bazak L., Buchumenski I., Porath H.T., Danan-Gotthold M., Knisbacher B.A., Eisenberg E., Levanon E.Y. (2015). Elevated RNA editing activity is a major contributor to transcriptomic diversity in tumors. Cell Rep..

[26] Han L., Diao L., Yu S., Xu X., Li J., Zhang R., Yang Y., Werner H.M., Eterovic A.K., Yuan Y. (2015). The genomic landscape and clinical relevance of A-to-I RNA editing in human cancers. Cancer Cell.

[27] Zuker M. (2003). Mfold web server for nucleic acid folding and hybridization prediction. Nucl. Acids Res..

[28] Reautschnig P., Vogel P., Stafforst T. (2016). The notorious RNA in the spotlight—Drug or target for the treatment of disease. RNA Biol..

