# Supplementary material for: Applying Human ADAR1p110 and ADAR1p150 for Site-Directed RNA Editing—G/C Substitution Stabilizes GuideRNAs against Editing
Source: Genes (Basel). 2017 Jan 14;8(1):34. doi: 10.3390/genes8010034 (PMC5295028; doi:10.3390/genes8010034)
Supplement: Supplementary file 1 [file genes-08-00034-s001.pdf]

Supplementary Materials: Applying Human ADAR1p110 and ADAR1p150 for Site-Directed RNA Editing—G/C Substitution Stabilizes GuideRNAs Against Editing

Madeleine Heep, Pia Mach, Philipp Reautschnig, Jacqueline Wettengel and Thorsten Stafforst

Gene	***Sequence (5' to 3')***	***Product size***
ADAR1	*fw.: GCATTTGAGGATGGACTACG*	*101 bp*
	rev.: TCCTTAGTCTTCCCGGATTG	
ADAR2	*fw.: CGGAGATCCTTGCTCAGATT*	*99 bp*
	rev.: CCCTCGCTCTGATTTCTGAA	
ß-actin	*fw.: CGGGACCTGACTGACTAC*	*91 bp*
	rev.: TAATGTCACGCACGATTTCC	

A. Primers for Qpcr.

Cell line	Mean C_T (β-actin)	Mean C_T (ADAR)	$2^{-\Delta C_T}$
ADAR2 -dox (control)	18.596	24.574	0.02
ADAR1 (p150) - dox	18.836	22.634	0.07
ADAR1 (p110) - dox	19.153	22.057	0.13
ADAR2 + dox (control)	18.597	19.026	0.74
ADAR1 (p150) + dox	18.821	19.587	0.59
ADAR1 (p110) + dox	18.584	19.290	0.61

B. Measured ct-values of all experiments. Values are averaged from three technical replicates. Calculation of relative expression levels from the Δct values for ADAR2 versus the housekeeping gene ß-actin. (relative expression = $2^{-\Delta ct}$, with $\Delta ct = ct(ADAR1/2) - ct(ß - actin)$).

Figure S1. qPCR analysis of ADAR expression in engineered cells. The relative amount of ADAR mRNA in 293T cells with a genomically integrated copy of ADAR controlled by a CMV tet-on promoter (integr.) was determined by quantitative real-time PCR (qPCR) after 72h (doxycycline induced expression of integr. ADAR). For this, RNA was extracted from cell lysates (RNeasy MinElute Kit, Qiagen, Hilden, Germany). After DNaseI digestion (NEB) and reverse transcription (high capacity cDNA reverse transcription kit, Applied Biosystems, Foster City, CA, USA), 20 ng cDNA was mixed with Fast SYBR Green Master Mix (Applied Biosystems) and analyzed by the 7500 Fast Real-Time PCR System (Applied Biosystems). (**A**) For determining gene expression, primers were designed for targeting ADAR1, ADAR2 and the housekeeping gene ß-actin; (**B**) qPCR of ADAR1, ADAR2 and the housekeeping gene was performed in triplicates. The table displays the mean values of the cycles where the fluorescence crosses the threshold of 0.35 for ADAR and 0.15 for ß-actin (ct values).

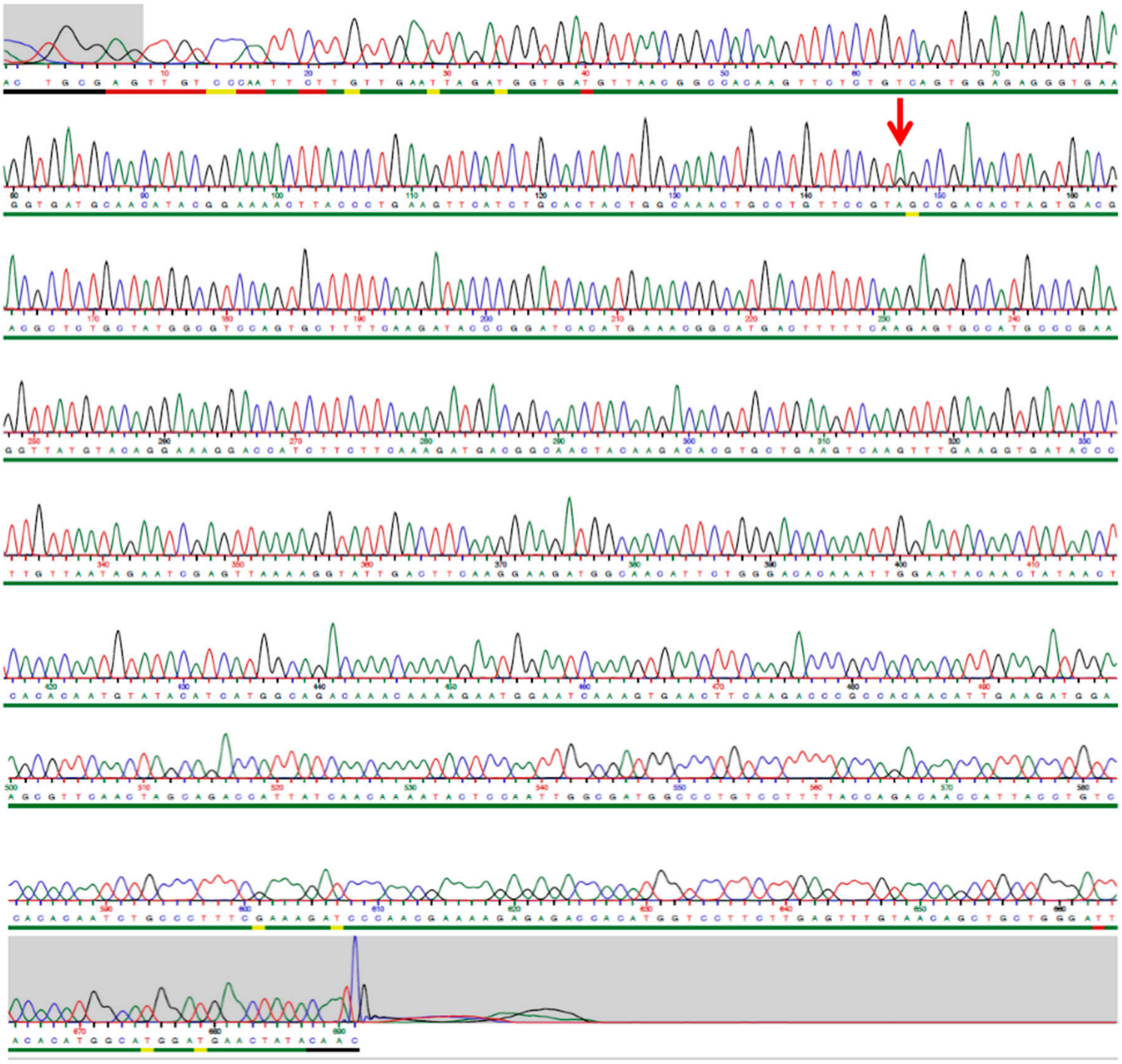

Figure S2. Sanger sequencing trace of editing in ADAR1p110-expressing cells. The target site is marked by a red arrow.

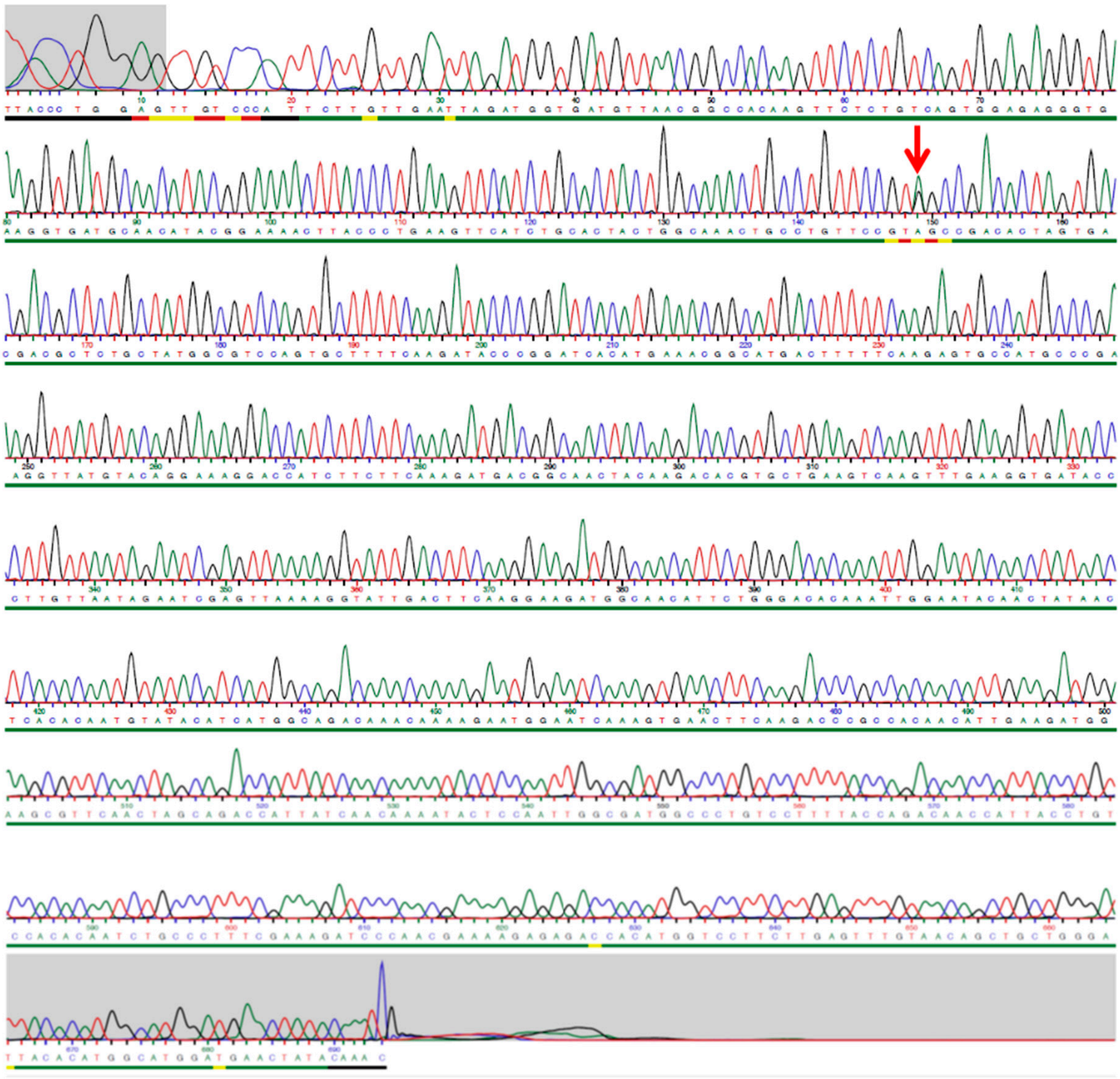

Figure S3. Sanger sequencing trace of editing in ADAR1p150-expressing cells. The target site is marked by a red arrow.

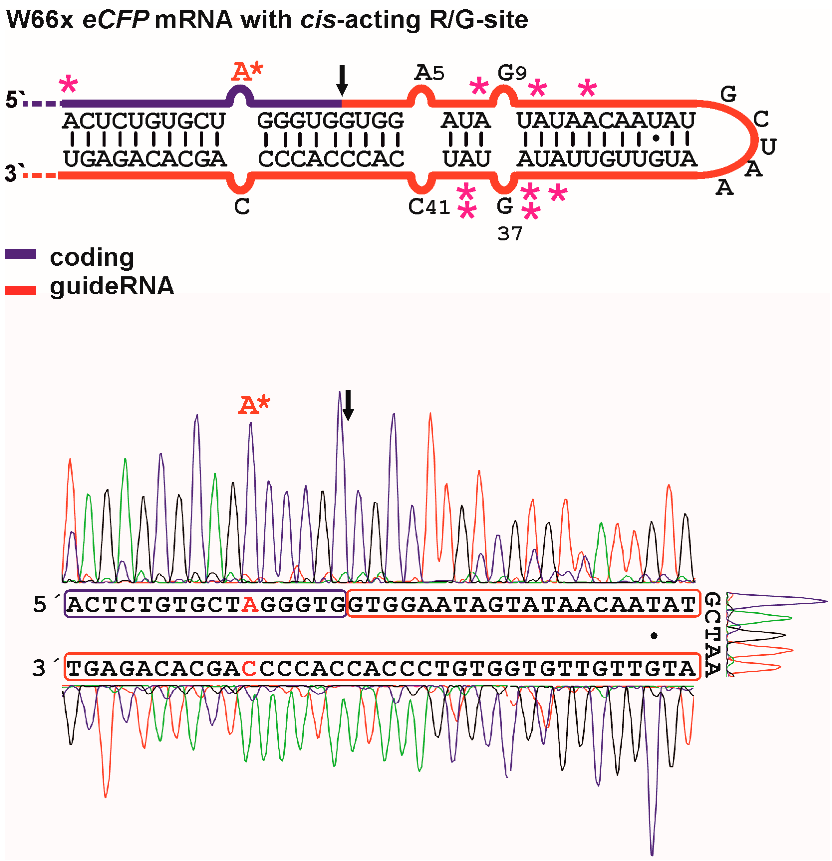

Figure S4. Defining auto-editing hotspots in cellular editing. The same construct as described in Figure 2 main text was used as an auto-editing probe inside the 293 cell. Cells were co-transfected in a 24-well format with a pcDNA3.1 vectors containing the transcript for editing (300 ng) and a pcDNA3.1 vector containing ADAR2 (300 ng). After 24 h, RNA was isolated and sequenced.

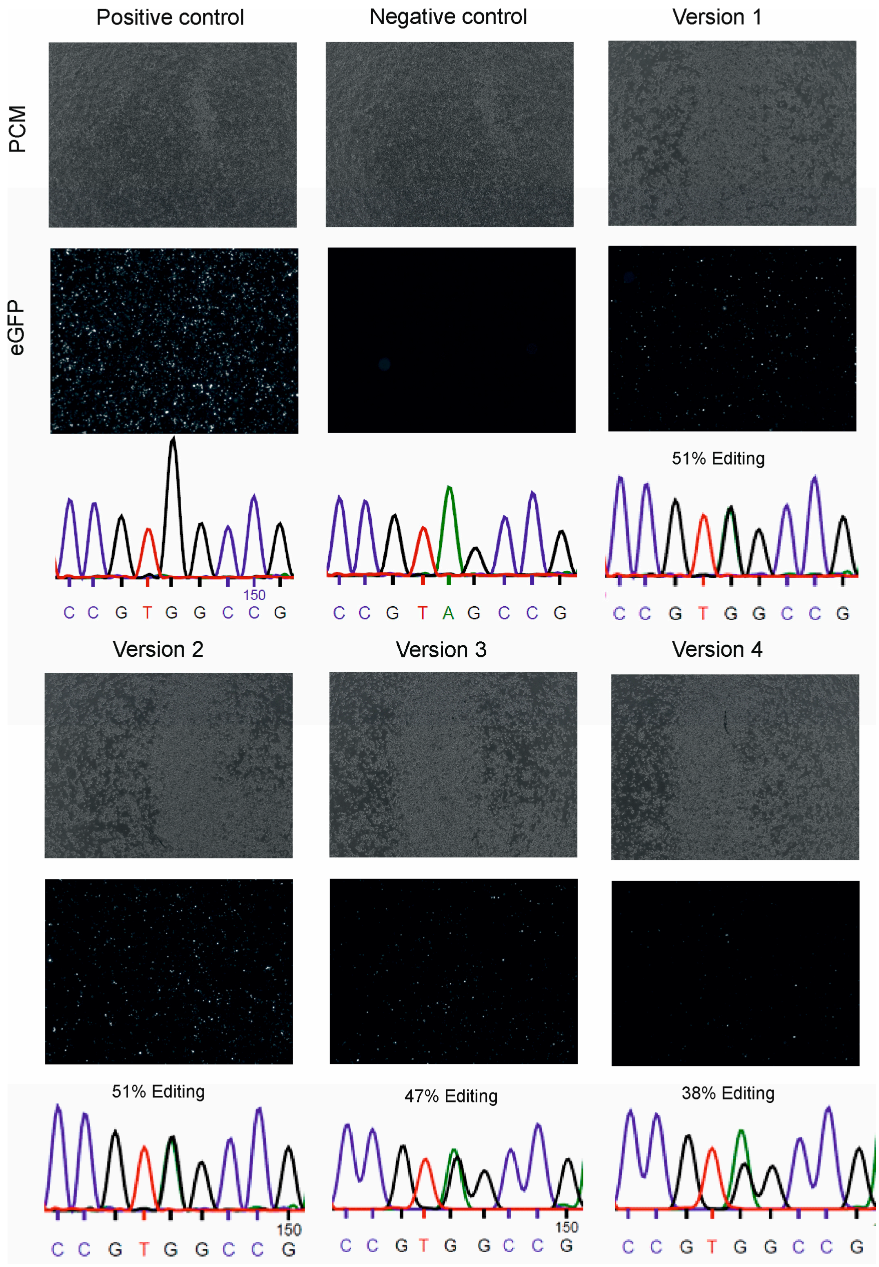

Figure S5. Fluorescence imaging data belonging to the experiment shown in Figure 3B, main text. Fluorescence images (50x magnification, 70 ms exposure for GFP) were taken 72 h after co-transfection with the respective gRNA (version 1 to 4, 1300 ng) and GFP W58X amber (300 ng). Transfection with 300 ng wild-type GFP and 300 ng GFP W58X amber served as positive and negative controls respectively. Sanger sequencing was performed after RNA isolation (NucleoSpin RNA Plus Kit, Macherey-Nagel, Dueren, Germany), reverse transcription and Taq-PCR. (eGFP = fluorescence channel, PCM = phase contrast microscopy).

© 2017 by the authors. Submitted for possible open access publication under the terms and conditions of the Creative Commons Attribution (CC-BY) license (http://creativecommons.org/licenses/by/4.0/).
